# Establishment and a comparative transcriptomic analysis of a male-specific cell line from the African malaria mosquito *Anopheles gambiae*

**DOI:** 10.1038/s41598-022-10686-y

**Published:** 2022-04-27

**Authors:** Elzbieta Krzywinska, Luca Ferretti, Jaroslaw Krzywinski

**Affiliations:** 1 Vector Molecular Biology Group, The Pirbright Institute, Pirbright, UK; 2 Big Data Institute, Nuffield Department of Medicine, University of Oxford, Old Road Campus, Oxford, OX3 7LF UK

**Keywords:** Transcriptomics, Non-model organisms

## Abstract

Cell lines allow studying various biological processes that may not be easily tractable in whole organisms. Here, we have established the first male-specific cell line from the African malaria mosquito, *Anopheles gambiae*. The cells, named AgMM and derived from the sex-sorted neonate larvae, were able to undergo spontaneous contractions for a number of passages following establishment, indicating their myoblast origin. Comparison of their transcriptome to the transcriptome of an *A. gambiae*-derived Sua5.1 hemocyte cells revealed distinguishing molecular signatures of each cell line, including numerous muscle-related genes that were highly and uniquely expressed in the AgMM cells. Moreover, the AgMM cells express the primary sex determiner gene *Yob* and support male sex determination and dosage compensation pathways. Therefore, the AgMM cell line represents a valuable tool for molecular and biochemical in vitro studies of these male-specific processes. In a broader context, a rich transcriptomic data set generated in this study contributes to a better understanding of transcribed regions of the *A. gambiae* genome and sheds light on the biology of both cell types, facilitating their anticipated use for various cell-based assays.

## Introduction

Insect cell lines represent an important in vitro tool for addressing a wide range of biological questions. Although not exact equivalents of cells in an organism, due to adaptations to growth in culture and often large genomic rearrangements occurring during establishment and subsequent passaging^1^, immortalised cells retain many normal physiological features, allowing studies of diverse processes related to immunity, biochemistry, as well as molecular, developmental and cell biology of the source organisms^2^.

More than 500 continuous cell lines representing various cell types have been established from over 100, mainly lepidopteran and dipteran, insect species^3,4^. Among them, thirteen named cell lines originated from the African malaria mosquito *Anopheles gambiae* (Supplementary Table S1), all derived from mixed-sex neonate larvae^5–7^. Despite high tissue complexity of the source material, all these mosquito cell lines were found to have hemocyte-like properties^7–10^.

The *A. gambiae* Sua5.1 hemocyte-like cell line^7,11^ has been extensively used in studies on mosquito immunity^12–19^. It was also used as an in vitro tool, for example, to test the functionality of the bi-partite GAL4-UAS expression system^20^, to explore the activity of putative constitutive promoters of the *A. gambiae* genes^21,22^, or to study fundamental developmental biology of *A. gambiae*^23,24^.

The Sua5.1 cells were found to be female-like, because they lack the Y chromosome and support cell-autonomous female-specific processes, including apparently fully functional sex determination molecular pathway, as manifested by the female-specific splicing of its terminal gene *doublesex* (*dsx*)^23^. These properties proved pivotal to exploration of the *A. gambiae* sex determination genes located upstream from *dsx*, facilitating identification of *Yob*, the primary sex determiner gene conferring maleness^23^, and of *fle*, a gene that controls both *dsx* splicing and suppression of dosage compensation in females^24^. However, the Sua5.1 cell line is not adequate as a tool for examining male-specific molecular interactions involving *Yob*.

To address this shortcoming, we established in the present study the first male-specific *A. gambiae* cell line (which we call AgMM) derived from neonate larvae of an *A. gambiae* strain whose transgenic progeny is exclusively male. Morphology of the cells and their propensity to form long multicellular contractile structures indicated that AgMM are composed primarily of myocytes. Analysis of the AgMM transcriptome and a comparison to the transcriptome of Sua5.1 cells supported this notion, because a number of genes expressed highly and uniquely in AgMM encode proteins involved in muscle development and function. The AgMM cells express *Yob* and a male-specific form of *dsx*, which indicates the presence of a functional sex determination pathway. In addition, a yet unknown dosage compensation machinery is active, as indicated by a comparable transcriptional output from the single X chromosome and the autosomes. Therefore, the AgMM cell line represents a valuable resource that will greatly facilitate molecular and biochemical studies on the molecular interactions of *Yob* and on the identification and analysis of the dosage compensation components in *A. gambiae*. More broadly, a rich transcriptomic data set generated in this study sheds light on the biology of both cell types, facilitating their future use in cell-based assays. In addition, it allows a better insight into the transcriptional landscape of the *A. gambiae* genome by revealing deficiencies of the current *A. gambiae* genome annotation and the need for its further improvement.

## Materials and methods

### Mosquito rearing

*A. gambiae s.s.* wild-type G3 strain and the transgenic strain hb:Yob-D generated using the G3 strain genetic background were reared at 28 °C and 80% humidity on 12 h:12 h light:dark cycle, according to the standard protocol^25^. Larvae were reared in plastic trays filled with 1 L of deionised water and fed with ground TetraMin tropical fish food flakes (Tetra). The adults were kept in BugDorm-4 cages (BugDorm) with access to 10% sucrose solution ad libitum. Females were fed through the Hemotek membrane feeder with a 1:1 mixture of time-expired human red blood cells and plasma sourced from a blood bank.

### Generation of the primary cell cultures

A compilation of modified protocols^7,26^ has been adopted in this study. Seventy two hours after blood-feeding, wild-type females crossed to males from the transgenic strain hb:Yob-D were allowed to lay eggs on water for one hour in the dark. An hour later the eggs were collected from the water surface with a bacteriological loop and transferred to a sterile Petri dish with a 0.1% aqueous benzalkonium chloride (Sigma) solution for surface-sterilization. The eggs were washed by vigorous pipetting for 4 min, after which benzalkonium chloride was removed and the eggs were washed twice in sterile water with 10X Penicillin–Streptomycin (GIBCO). The subsequent steps were made in aseptic conditions whenever possible. Floating eggs were transferred with a loop to a Petri dish containing sterile water, then covered, and left undisturbed at 26 °C. After 36 h, the eggs were agitated by gentle shaking of the dish to promote synchronous larval hatching. Within 1–2 h after hatching, larvae were sorted under the fluorescence microscope, and approximately 100 transgenic individuals (which in the hb:Yob-D strain are exclusively males) were transferred in a small amount of water to a 1.5 ml Eppendorf tube placed vertically on ice. After the larvae became immobile and sank to the bottom, the water was removed, and a few drops of 0.25% trypsin solution were added to the tube. The larvae suspended in the trypsin solution were transferred to a Petri dish and each cut up into 3–4 pieces with a scalpel. The larval pieces were then transferred to 1 ml of 0.25% trypsin solution in an Eppendorf tube and incubated at 35 °C for 10 min. Subsequently, the content of the tube was mixed by moderately vigorous pipetting using a pipette tip cut at the end, and centrifuged at 125*g* for 10 min, after which the supernatant was removed, the pellet was washed twice in 1 ml of PBSA (Thermo Fisher) containing 10 × Penicillin/Streptomycin, with centrifugation as above after each wash. After removal of the supernatant, pellet was resuspended in 1 ml Schneider’s medium (Lonza) supplemented with 20% FBS/Penicillin–Streptomycin (300 units/ml–300 µg/ml) and Fungizone (2.5 µg/ml) (GIBCO). The resulting tissue suspension was transferred to a well of a 12-well plate and topped up with 1 ml of complete culture medium. Cultures were incubated at 28 °C undisturbed. After a week, half of the medium was gently removed from the top and replaced with a fresh medium containing 20% FBS and Pen/Strep (100 units/ml and 100 µg/ml); similar changes of half of the medium were then conducted weekly. After the first week, cultures were regularly examined for cell growth using an inverted microscope.

### Establishment of a cell line

When substantial cell growth was observed, with up to approximately half of the well covered with cell patches, re-seeding was done to ensure smoother distribution of cells. The medium with any floating tissue clumps were transferred to a tube, the clumps allowed to settle, and top half volume of the medium was aspirated and discarded. Then, half volume of fresh medium was added to the original well and pipetted in an effort to dislodge and break up clumps of cells, after which the sedimented cells with the half volume of old medium were returned to the well, and the cells were left for further growth. With further growth observed, the medium was pipetted in an attempt to dislodge and break clumps of cells and the cells were subcultured. Half of the cell suspension was transferred to a new well, and half remained in the original well; an equal volume of fresh medium was then added to both wells and the cells were left for growth. The procedure was repeated, after which cells from one daughter well were split to a cell culture flask, to which fresh medium was added. With the evidence of sufficient growth in the flask, cells were dislodged using a cell scraper and by pipetting, and then split into a daughter flask, similar to subculturing procedure in wells. All subsequent passages were conducted in flasks.

### Cryopreservation and reconstitution of the cells

On passage 15, the AgMM cells grown in a T75 flask were dislodged using scraper, centrifuged at 150×*g* for 5 min at room temperature and after discarding supernatant, resuspended in a cryopreservation medium containing 5% DMSO, 45% FCS, and Schneider’s medium. Cells dispensed into cryotubes were placed into a Mr. Frosty cooling system (Nalgene) and stored at − 80 °C for 24 h before transferring to the liquid nitrogen. Cryopreserved cells were resuscitated by thawing the cryovial content in a 37 °C water bath and transferring the content, before complete thawing, to a complete medium (Schneider’s medium supplemented with 20% FBS/Penicillin–Streptomycin (100 units/ml–100 µg/ml) warmed up to 27 °C, followed by a subsequent spinning down at 150×*g* for 5 min and aspirating the supernatant to remove DMSO. The pelleted cells were resuspended in 10 ml of warm complete medium, transferred to a T25 flask, and were incubated at 27 °C until desired confluency.

### Transfection of the cell line

The AgMM cells were seeded into a 24-well plate 2–3 days prior to transfection, and transfection experiments were performed when their confluency reached about 80%. The cells were transfected with 1 μl of Lipofectamine 3000 Reagent (Life Technologies), 1 μl of P3000 Reagent and 0.3 μg of a plasmid DNA per well, according to a manufacturer’s protocol. The plasmid contained the eGFP open reading frame under the control of the *A. gambiae *polyubiquitin promoter, allowing evaluation of transfection efficiency using fluorescence microscopy.

### PCR and RT-PCR

Presence of the Y chromosome in the AgMM cells was tested by PCR using primers 124678F2 (5′-TTTGAGCATGTGTTTAAAGG-3′) and 124678R2 (5′-AGGTTTTCCCGAGTACAAT-3′). The RNA was isolated from the cells using with PureLink RNA Micro Scale kit (Invitrogen) according to the manufacturer's protocol. Transcription levels of Yob were evaluated through RT-PCR using primers T7YobF (5′-TAATACGACTCACTATAGGGATGTTTGTTTTGTATGTGTCG-3′) and Yob_endR (5′-GATATTTTAATTGTTTTTATTCGAGCGG-3′), and the SuperScript III One-Step RT-PCR System with Platinum® Taq DNA Polymerase (Invitrogen) according to the manufacturer’s guidelines. RT-PCR was also used to examine the splicing pattern of the *dsx* using primers dsxF2 (5′-CCAGAACCTGTAAATCTCCTAC-3′) and dsxR5m (5′-GATGACTTCACCACCGCTTC-3′). A fragment of the ribosomal protein S7 gene mRNA was amplified using primers S7F (5′-TGCTGCAAACTTCGGCTAT-3′) and S7R (5′-CGCTATGGTGTTCGGTTCC-3′), to serve as an internal control of equal sample loading in each RT-PCR experiment. Identity of the RT-PCR products with the target sequences was confirmed by sequencing.

### Collection of RNA samples and RNA-seq analysis

Total RNA was extracted from growing cells (for AgMM harvested at passage 11, after the cells lost the ability to contract) using Trizol (Invitrogen) with PureLink RNA Micro Scale kit (Invitrogen) and its integrity checked using TapeStation (Agilent). Triplicate RNA samples from AgMM and Sua5.1 cell lines were processed to generate sequencing libraries. The TruSeq library preparation protocol (Illumina) was followed by 150 bp paired-end sequencing using NovaSeq 6000 sequencing system (Illumina). Trimmomatic version 0.30^27^ was used to remove sequencing adapters used in library generation and to remove low-quality reads. To ensure the success of the filtering step, the quality of the reads was subsequently assessed with FastQC version 0.10.1^28^. Read counts matrices were generated with two approaches. First, the reads were mapped to the *A. gambiae* PEST genome using STAR v. 2.7.3a^29^ and the resulting BAM files were used in featureCounts from the package Subread v. 1.6.4^30^ for read summarization. Second, the reads were pseudo-aligned to the A. gambiae transcriptome genebuild AgamP4.12 using Kallisto v0.46^31^. Both approaches yielded very similar results. Transcripts per kilobase million (TPM) value was quantified for each transcript and averaged across multiple replicates of the same sample. Differential expression analysis was performed on raw read counts using DESeq2^32^, after removing genes with less than 10 reads across all samples. Genes were considered as significantly differentially expressed when a higher than two-fold difference in expression level (log_2_(FC) >|1|) was observed between the cell lines and the adjusted p value was below 0.0001 (Wald test, with Benjamini–Hochberg correction).

The PANTHER v16.0^33^ was used to identify GO terms enrichment in genes expressed specifically in either AgMM or Sua5.1 cells against a background of all genes expressed above a threshold of 0.5 TPM in a given cell line. Significance of the statistical overrepresentation was calculated using Fisher’s Exact test with False Discovery Rate correction.

For the analysis of dosage compensation, we compared the median expression levels from the X chromosome and the autosomes in both AgMM and Sua5.1 cells at a range of thresholds of minimum expression levels, ranging from TPM = 0, which includes both active and nonactive genes, and TPM = 40, in which only highly expressed genes were taken into account. To evaluate significance of differences in expression between chromosomes, we computed the 95% confidence intervals for the X:A ratios of median expression by bootstrapping using R^34^. We performed 10,000 bootstrap replicates, stratifying by chromosome and sample.

## Results and discussion

### Establishment of a male-specific *A. gambiae* cell line

**Figure 1:**
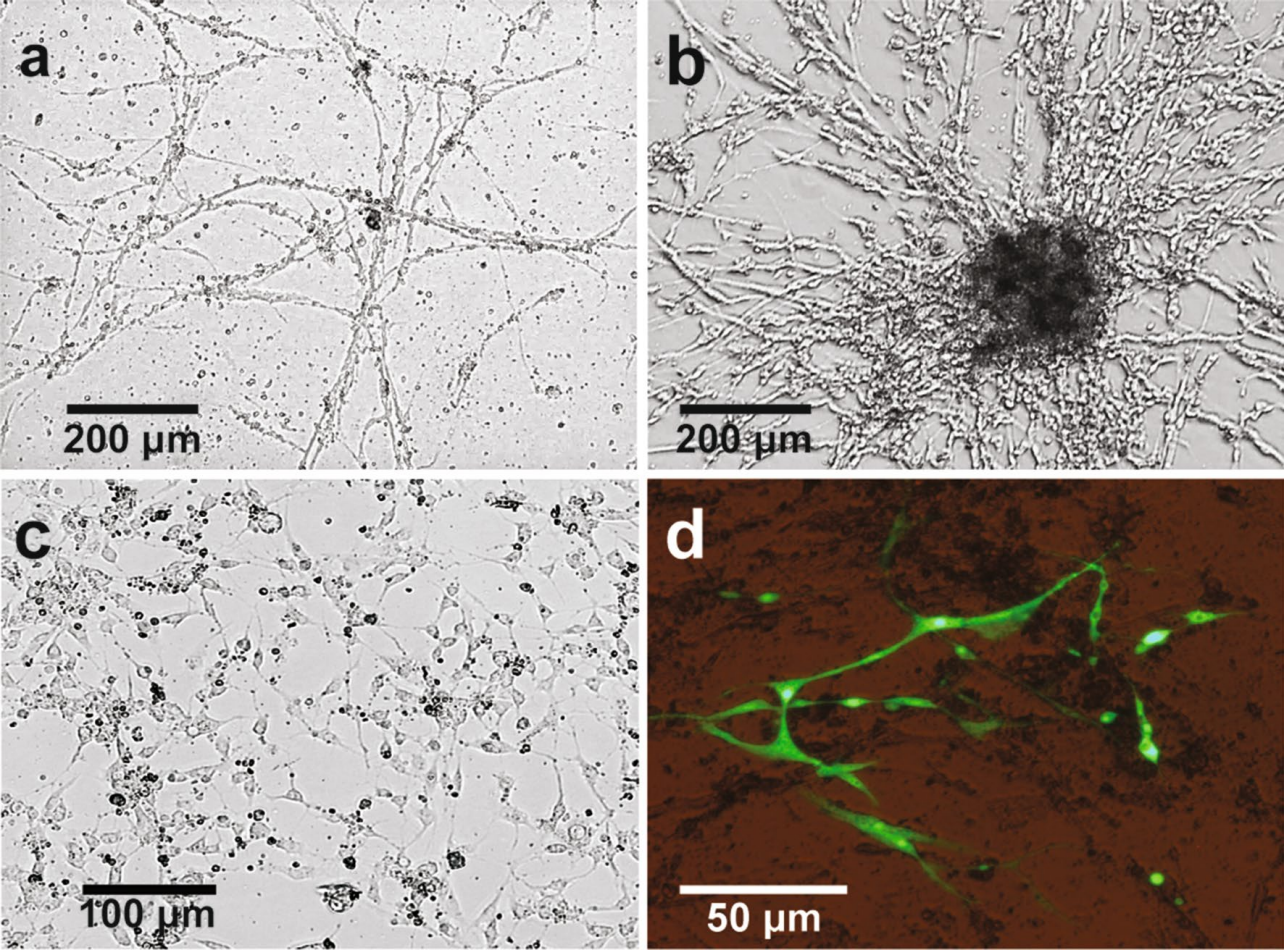
Light micrographs of the AgMM cells (**a**) at passage 4, 4 days after seeding; (**b**) at passage 4, 14 days after seeding, with the multi-layered node; (**c**) at passage 12, 4 days after seeding; (**d**) at passage 12, 48 h post-transfection with a plasmid containing an expression cassette with eGFP under the control of the *A. gambiae* polyubiquitin promoter.

Mosquito male and female neonate larvae are morphologically indistinguishable. Therefore, to obtain a pure collection of neonate male larvae as a starting material for the primary culture, we used a transgenic *A. gambiae* line hb:Yob-D, in which females carrying the transgene do not survive beyond embryonic stage due to issues with abnormally activated dosage compensation. In result, crosses between wild-type females and hb:Yob-D males produce wild-type males and females, as well as transgenic males tagged with the EGFP marker. Using a fluorescence microscope, we collected two batches of approximately 100 transgenic neonate male larvae that were separately processed to generate two primary cell cultures. Initially, both cultures contained fragments of viable twitching tissue, larval pieces with attached growing clumps of cells, as well as highly elongated, slowly growing adherent spindle-shaped cells extending from the larval pieces. The growth was arrested in one of the cultures after approximately three months, whereas in the unaffected culture the adherent elongated cells became predominant. These cells grew often in clumps and produced long, thread-like, anastomosing cytoplasmic protrusions (Fig. 1a). In addition to the network pattern and fibre-like aggregates, the cells formed multi-layered nodes (Fig. 1b), and after reaching a certain cell mass, began spontaneous persistent contracting (Supplementary File 1), indicative of a myoblast origin of the cell line. Accordingly, the cell line was named AgMM (*A. gambiae* male myocytes). The cells were subcultured and subjected to successive passaging, during which the propensity for spontaneous contractions started to diminish at certain point and ceased from passage 10 onwards, which was also associated with a loss of the highly elongated fiber-like cell morphology (Fig. 1c). Evidence from a study on *Manduca sexta* myogenic cells indicated that an elongated, spindle-like morphology and the capacity to fuse and form contractile myotubes, can be achieved by myocytes only in the presence of neurons^35^. Therefore, it is plausible that initially the AgMM cell line contained an admixture of neural cells, which were subsequently lost from the culture, likely because of their lower mitotic potential than that of the myocytes.

At the time of submission of the paper, the cells have undergone over 20 passages, as well as cryopreservation, followed by a subsequent reconstitution and passaging. The AgMM cells are strongly adherent to both the flask surface and to each other, and a cell scraper must be used for their efficient dislodging, in contrast to the Sua5.1 cells, which can be relatively easily dislodged through tapping the side of the flask and separated from each other by gentle pipetting.

### Molecular characterisation and manipulation of the AgMM cells

**Figure 2:**
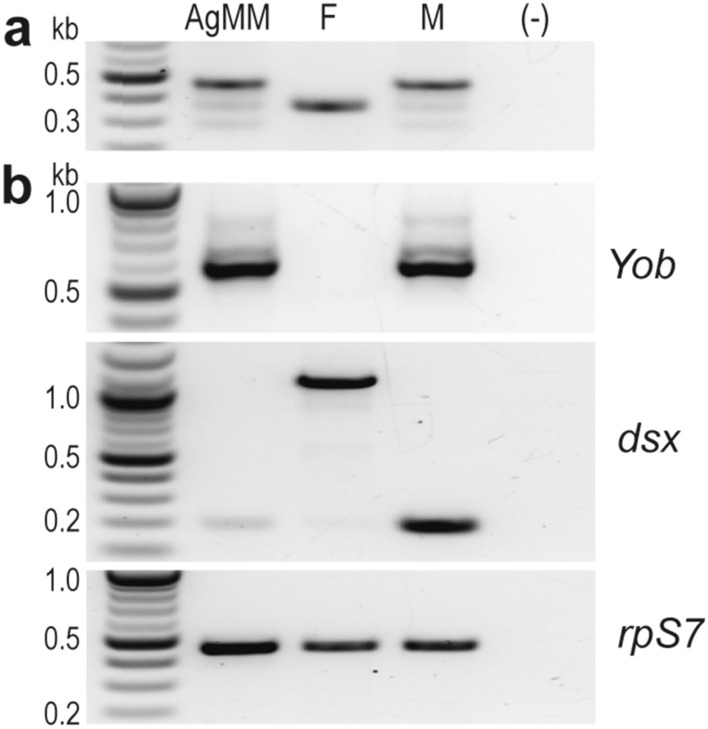
Molecular characterisation of the AgMM cell line. (**a**) Sexing PCR shows male origin and presence of the Y chromosome in the AgMM cells. (**b**) RT-PCR analysis of transcription and splicing pattern of the sex determination genes *Yob* and *dsx*. F, female, and M, male samples were used for comparison; (–), no template control. Ribosomal protein S7 (rpS7) transcript levels were used as a gel loading control.

We confirmed the male origin and presence of the Y chromosome in the AgMM cells (Fig. 2a) by conducting molecular karyotyping PCR^23^. We also confirmed, through RT-PCR, that the male determiner gene *Yob* is expressed and thus may regulate the male-specific molecular processes (Fig. 2b). This is indeed the case, because *dsx*, although expressed at very low levels, is male-specifically spliced (Fig. 2b), which indicates that the cells support a functional male sex determination pathway. Moreover, dosage compensation, another male-specific process, is ongoing in the AgMM cells (see below).

Using a lipid-based transfection reagent and a plasmid with an expression cassette driving ubiquitous eGFP expression in the *A. gambiae* cells, we demonstrated that the AgMM cells are amenable to transfection (Fig. 1d), with the efficiency reaching approximately 20%. Higher proportion of transfected cells could possibly be achieved after optimisation of transfection conditions, or by establishing a stable transfection^36^.

We conducted cell cryopreservation and reconstitution experiments to evaluate the amenability of the AgMM cells to long-term storage conditions. After storage in both − 80 C and in the vapor phase over liquid nitrogen for a minimum of three months the cells did not lose viability or the ability to proliferate, which demonstrates that they can be conveniently kept frozen and reconstituted on demand.

### Expression differences between AgMM and Sua5.1

We performed RNA-seq analysis to gain an insight into the genome-wide gene expression in the AgMM cells and, for comparative purposes, in the Sua5.1 cells. The triplicate sequencing libraries were sampled, on average, to the depth of 72.3 and 89.7 million 150 bp paired-end reads, of which 91% and 89% were uniquely mapped for AgMM and Sua5.1, respectively (Supplementary Table S2).

**Figure 3:**
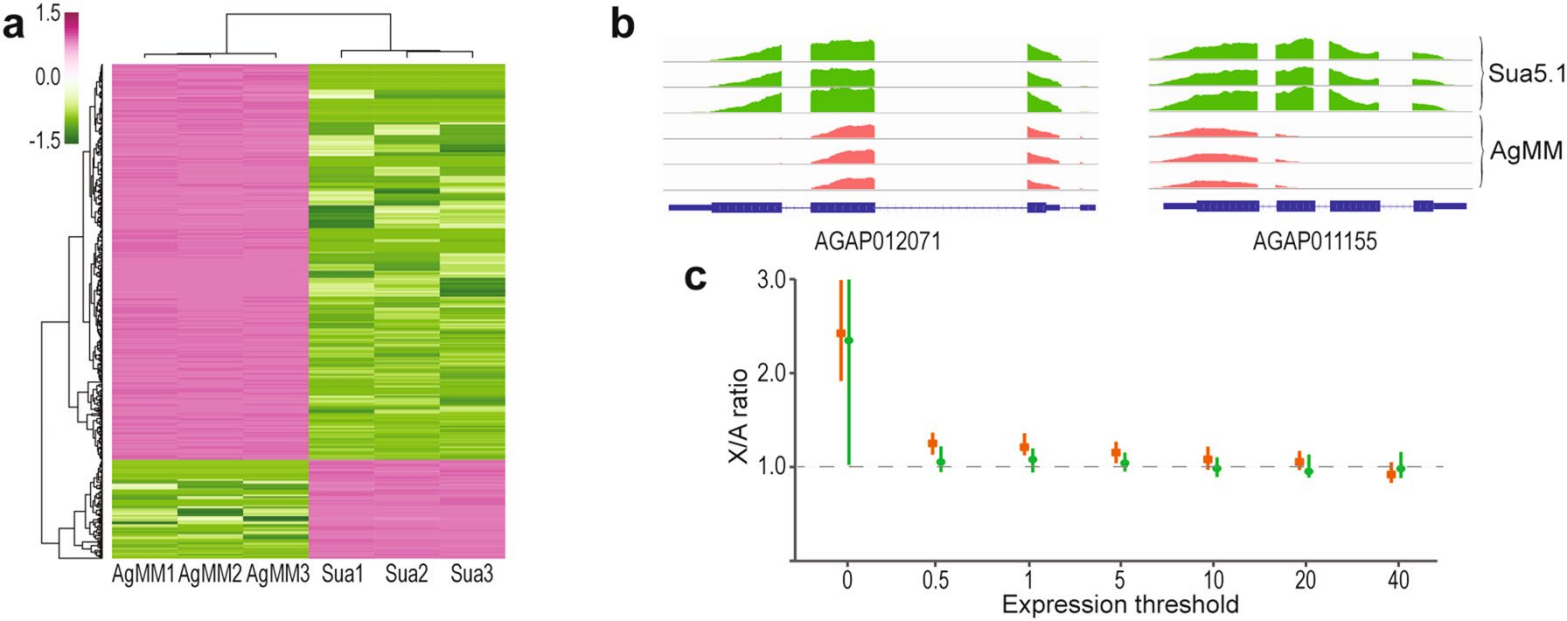
Analysis of expression in the AgMM and Sua5.1 cell lines. (**a**) A heat map of the top 500 genes most variable between the AgMM and Sua5.1 cells. Dark pink indicates the most positive z-score, while dark green indicates the most negative z-score. (**b**) Examples of genes with alternative transcription between the cell lines. (**c**) Analysis of dosage compensation in the AgMM cells. Chromosome-wide ratios of median expression from the X chromosome and the autosomes in the AgMM (red) and, for comparison, Sua5.1 (green) cells as a function of minimum expression (TPM) levels. For each threshold, dots represent the median and vertical bars indicate the 95% confidence intervals from bootstrap.

The expression profiles of the two cell lines dramatically differ, likely reflecting distinct physiology and function of the respective founder cells. By applying a low threshold (0.5 TPM) to take into account rare transcripts, we identified 8007 and 7318 genes expressed in the AgMM and Sua5.1 cells, respectively. Both cell types shared 6929 expressed genes. Some of that expression is likely modulated by adaptation of the cells to growth in vitro^37^; however, the extent of these modifying effects remains unknown. Differential expression analysis revealed 2354 genes in AgMM and 1567 genes in the Sua5.1 cells when a stringent cutoff was applied (an adjusted *p* value < 0.0001 and at least a two-fold higher expression level in one cell line compared to the other cell line) (Fig. 3a).

To characterise cell line-specific molecular signatures, we focused on the genes expressed in only one cell line (with zero mapped reads in at least one replicate from the other cell line) and conducted their functional analysis. When ranked by the expression levels, the top 250 AgMM cell line genes (expression ranging from 2700.7 to 2.2 TPM) were enriched for involvement in muscle function and organisation, including orthologs of *Drosophila wupA*, *Tm2*, *Zasp52*, and *Zasp66* associated with actin filament-based process and myofibril GO terms, corroborating the presumed myoblast origin of the AgMM cells (Supplementary Table S3). Among very highly expressed were also muscle-related orthologs of *Drosophila sing*, *twi*, and *Prm*. Expression of *twi* has been regarded as a molecular marker for embryonic stem cell-like adult muscle precursor (AMP) cells^38^. *twi* is a transcription factor, whose expression ceases with the beginning of the AMPs differentiation, which in turn coincides with an onset of expression of a number of terminal differentiation and muscle identity genes, including *Tm2*, *mhc*, and *gel*^39,40^; their orthologs are highly expressed in the AgMM cells. A time-course analysis of transcriptional changes during immortalisation of *Drosophila* embryonic myocytes revealed a small cluster of genes with an expression profile highly correlated to that of *twi*^40^, among them *kon* and *nvy*, *A. gambiae* orthologs of which have moderate to high expression in AgMM cells. In addition to the muscle-related GO terms, the cell line-specific expression in AgMM cells was characterised by a significant overrepresentation of genes corresponding to secreted cellular components, proteolysis, and chitin metabolism. The latter GO term seems unexpected; however, the function of the genes involved is unclear. Although they all encode GH-18 domain characteristic of chitinases, the domain is incomplete in two genes, and two other genes in this category represent chitinase-like imaginal disc growth factors *Idgf2* and *Idgf4*. IDGFs lack chitinase activity and, instead, are involved in cell proliferation and differentiation in insects^41,42^.

In case of the Sua5.1 cells, the functional analysis involved the top 118 genes (expression ranging from 1681.8 to 1.0 TPM), which were enriched for GO terms related to cellular carbohydrate metabolism and chemosensory perception (Supplementary Table S4). Overrepresentation of chemosensory genes is consistent with their enrichment in oenocytoid hemocytes isolated from perfused haemolymph of adult *A. gambiae* females^43^.

For the selected set of 250 and 118 genes, we compared the transcript levels in the cells and in the sexed whole *A. gambiae* 4th instar larvae and pupae^44^. Expression of the majority of the compared genes was significantly higher in the cells than in the whole mosquitoes, which appears to support their cell line specificity (Supplementary Table S5). In these cases, a single cell type highly enriched in in vitro culture produces a prominent expression pattern; however, because in the whole mosquitoes the given cell type constitutes a small proportion of all cells, their transcriptomic signal is swamped by a plethora of transcripts from all other tissues. Comparison of expression in the *Drosophila melanogaster* cell lines and whole flies led to similar observations^37^. Some highly expressed putative cell line-specific genes in our study have no clear homologs in *Drosophila* or other non-culicid insects, which suggests their likely origin in mosquito ancestors. Several of these genes may be non-coding, because they have a short predicted open reading frame that is poorly conserved even among relatively closely related members of the genus *Anopheles*. Finally, a number of putative cell-specific genes have unknown function; thus, identification of their cell specificity per se contributes to their functional annotation.

Although we have not specifically considered alternative transcript isoforms here, we noticed examples of the cell line-limited alternative transcription (Fig. 3b), which undoubtedly represents a considerable source of transcript variation between the studied cell lines. Moreover, we have found a number of incorrect, incomplete, or missing annotations, also for genes with a cell line-limited transcription, revealing deficiencies of the current *A. gambiae* genome annotation and the need for its further improvement.

### Dosage compensation

Dosage compensation (DC) is a process that balances gene expression from the male monosomic X chromosome and from the diploid set of autosomes. In *A. gambiae*, analysis of RNA-seq data from whole-body male and female larvae and pupae revealed that in soma DC relies on a roughly twofold upregulation of the X chromosome expression in males to the levels of expression from autosomes and to the levels of expression from both X chromosomes in females^44^. DC is absent in *A. gambiae* male germline^45^ and, because it has not been otherwise studied below the organismal level, it remains unclear whether DC operates in *all* somatic tissues or in in vitro cultures. Consistent with the DC definition, the appropriate approach to testing for DC is a comparison of a chromosome wide expression output from the X chromosome and the autosomes (cf.^44^ and references therein for a discussion on pitfalls of different approaches to dosage compensation analysis). Whereas only transcriptionally active genes should be used for such comparisons, distinguishing genes expressed at very low levels from transcriptional background noise may not be straightforward. Therefore, we tested for DC in the AgMM cell line by comparing the median values of transcription from the X chromosome and from the autosomes (X:A) at increasing levels of expression threshold (TPM from 0 to 40). Apart from 0 TPM threshold, in which both active and non-active genes are included, the X:A ratios were close to 1.0 across the whole range of thresholds, indicative of complete DC, with a potential slight overcompensation when genes with low levels of transcription are considered (Fig. 3c). A similar comparison of a chromosome-wide transcriptional magnitude in Sua5.1 cells showed that transcription from both X chromosomes and the autosomes was also approximately equal (X:A around 1.0), consistent with the expectation (Fig. 3c).

The molecular interactions involved in DC in *Anopheles* remain unknown. In insects, regulation of DC has been deciphered only in *Drosophila*, in which male-specific lethal (MSL) complex consisting of at least five proteins and two noncoding RNAs binds specifically the single X chromosome in males and, through histone H4 lysine 16 acetylation (H4K16ac), leads to a two-fold upregulation of the X-linked genes^46–48^. Assembly of the DC complex is male-specific, because in females, translation of MSL2, a key protein of the complex, is blocked by a female-specific protein SXL involved also in sex determination. A recent study demonstrated that the DC control in *Anopheles* is achieved by a completely different mechanism not employing MSL2 or H4K16ac^49^. The availability of the AgMM cell line may greatly facilitate identification and validation of the DC machinery components in *Anopheles*. In particular, a relative ease of transcription manipulation through transfection, combined with a short time investment required, and a practically unlimited supply of male cells should enable a rapid progress in the biochemical analyses of molecular interactions involved not only in DC but also in male sex determination.

## Supplementary Information

Supplementary Information 1.

Supplementary Information 2.

Supplementary Video 1.

## Data Availability

The RNA-seq data have been deposited in the European Nucleotide Archive with the accession number PRJEB50856. The AgMM cell line is available from the corresponding author upon request.
