# Supplementary material for: Establishment and a comparative transcriptomic analysis of a male-specific cell line from the African malaria mosquito Anopheles gambiae
Source: Sci Rep. 2022 Apr 27;12:6885. doi: 10.1038/s41598-022-10686-y (PMC9046191; doi:10.1038/s41598-022-10686-y)
Supplement: Supplementary file 1 — Supplementary Information 1. [file 41598_2022_10686_MOESM1_ESM.pdf]

Supplementary File

Establishment and a comparative transcriptomic analysis of a male-specific cell line from the African malaria mosquito *Anopheles gambiae*

Elzbieta Krzywinska[1], Luca Ferretti[2], and Jaroslaw Krzywinski[1*]

[1]Vector Molecular Biology Group, The Pirbright Institute, Pirbright, UK
[2]Big Data Institute, Nuffield Department of Medicine, University of Oxford, Old Road Campus, Oxford OX3 7LF, UK

*jaroslaw.krzywinski@pirbright.ac.uk

Supplementary Table S1. Described *An. gambiae* cell lines and their origin (*A. gambiae* strain and tissue).

Name	Origin	Reference
Mos. 55	Unknown strain neonate larvae	Marhoul and Pudney 1972
LSTM-AG-55	Unknown strain neonate larvae	Pudney *et a*l. 1979
4a-2	4a r/r neonate larvae	Müller *et a*l. 1999; Vizioli *et al*. 2000
4a-2s4	4a r/r neonate larvae	Müller *et al*. 1999; Knecht *et al*. 2003
4A-3A	4a r/r neonate larvae	Müller *et al*. 1999
4A-3B	4a r/r neonate larvae	Müller *et al*. 1999
L3-5	L3-5 neonate larvae	Müller *et al*. 1999; Vizioli *et al*. 2000
L3-5-5	L3-5 neonate larvae	Müller *et al*. 1999; Knecht *et al*. 2003
Sua1B	Suakoko 2La neonate larvae	Dimopoulos *et al*. 1997
Sua1.1	Suakoko 2La neonate larvae	Müller *et al*. 1999; Knecht *et al*. 2003
Sua 4.0	Suakoko 2La neonate larvae	Müller *et al*. 1999; Catteruccia *et al*. 2000
Sua5.1	Suakoko 2La neonate larvae	Müller *et al*. 1999; Catteruccia *et al*. 2000
Sua5B[1]	Suakoko 2La neonate larvae	Müller *et al*. 1999; Rasgon *et al*. 2006

[1]Split from Sua1B cell line.

References

Catteruccia F, Nolan T, Blass C, Müller HM, Crisanti A, Kafatos FC, Loukeris TG. 2000. Toward Anopheles transformation: Minos element activity in anopheline cells and embryos. *Proc. Natl. Acad. Sci. USA* **97**: 2157-2162.

Dimopoulos G, Richman A, Müller HM, Kafatos FC. 1997. Molecular immune responses of the mosquito Anopheles gambiae to bacteria and malaria parasites. *Proc. Natl. Acad. Sci. USA* **94**: 11508-11513.

Knecht W, Petersen GE, Sandrini MP, Sondergaard L, Munch-Petersen B, Piskur J. 2003. Mosquito has a single multisubstrate deoxyribonucleoside kinase characterized by unique substrate specificity. *Nucleic Acids Res.* **31**: 1665-1672.

Marhoul Z, Pudney M. 1972. A mosquito cell line (MOS. 55) from *Anopheles gambiae* larvae. *Trans. R. Soc. Trop. Med. Hyg.* **66**: 183-184.

Müller HM, Dimopoulos G, Blass C, Kafatos FC. 1999. A hemocyte-like cell line established from the malaria vector Anopheles gambiae expresses six prophenoloxidase genes. *J. Biol. Chem.* **274**: 11727-11735.

Pudney M, Varma MGR, Leake CJ. 1979. Establishment of cell lines from larvae of culicine (Aedes species) and anopheline mosquitoes. *TCA Manual* **5**: 997-1002.

Rasgon JL, Ren X, Petridis M. 2006. Can Anopheles gambiae be infected with Wolbachia pipientis? Insights from an in vitro system. *Appl. Environ. Microbiol.* **72**: 7718-7722.

Vizioli J, Bulet P, Charlet M, Lowenberger C, Blass C, Müller HM, Dimopoulos G, Hoffmann J, Kafatos FC, Richman A. 2000. Cloning and analysis of a cecropin gene from the malaria vector mosquito, Anopheles gambiae. *Insect Mol. Biol.* **9**: 75-84.

Suppmentary Table S2. Summary of RNA-seq data.

Sample	Total reads	Uniquely mapped	% Uniquely mapped
AgMM_1	75,030,521	68,854,029	91.77
AgMM_2	75,132,798	68,901,217	91.71
AgMM_3	66,837,874	61,482,139	91.98
Sua_1	87,328,202	77,839,152	89.13
Sua_2	79,253,031	70,918,700	89.48
Sua_3	102,483,285	91,286,442	89.07

Supplementary Table S3. GO overrepresentation analysis of top 250 genes (ranked by expression level) in the AgMM cells and lacking expression in the Sua5.1 cells.

GO ID	Term	Reference	Observed	Expected	FDR
	Cellular Component				
GO:0031941	filamentous actin	3	3	0.09	1.78E-02
GO:0030017	sarcomere	10	4	0.3	2.10E-02
GO:0005884	actin filament	12	4	0.37	3.51E-02
GO:0030016	myofibril	13	4	0.4	4.33E-02
GO:0030312	external encapsulating structure	17	5	0.52	1.58E-02
GO:0031012	extracellular matrix	17	5	0.52	1.51E-02
GO:0005615	extracellular space	72	14	2.19	1.77E-05
GO:0005576	extracellular region	118	21	3.59	5.92E-08
	Biological Process				
GO:0006032	chitin catabolic process	10	6	0.3	1.49E-03
GO:0006030	chitin metabolic process	10	6	0.3	1.35E-03
GO:0006026	aminoglycan catabolic process	13	7	0.4	9.45E-04
GO:1901072	glucosamine-containing compound catabolic process	12	6	0.37	2.35E-03
GO:1901071	glucosamine-containing compound metabolic process	12	6	0.37	2.19E-03
GO:0046348	amino sugar catabolic process	13	6	0.4	2.93E-03
GO:0006040	amino sugar metabolic process	15	6	0.46	4.70E-03
GO:0006022	aminoglycan metabolic process	19	7	0.58	2.13E-03
GO:1901136	carbohydrate derivative catabolic process	29	7	0.88	1.24E-02
GO:0030036	actin cytoskeleton organization	48	8	1.46	2.89E-02
GO:0030029	actin filament-based process	49	8	1.49	3.17E-02
	Molecular Function				
GO:0008061	chitin binding	13	6	0.4	2.31E-02
GO:0004252	serine-type endopeptidase activity	41	8	1.25	3.77E-02
GO:0008236	serine-type peptidase activity	46	8	1.4	5.12E-02
GO:0017171	serine hydrolase activity	46	8	1.4	4.39E-02

Supplementary Table S4. GO overrepresentation analysis of top 118 genes (ranked by expression level; cutoff: 1 TPM) in the Sua5.1 cells and lacking expression in the AgMM cells.

GO ID	Term	Reference	Observed	Expected	FDR
	Cellular Component				
GO:0043025	neuronal cell body	8	3	0.13	4.88E-02
GO:0044297	cell body	8	3	0.13	4.47E-02
	Biological Process				
GO:0019521	D-gluconate metabolic process	6	4	0.1	4.90E-03
GO:0019520	aldonic acid metabolic process	6	4	0.1	4.41E-03
GO:0046177	D-gluconate catabolic process	6	4	0.1	4.01E-03
GO:0046176	aldonic acid catabolic process	6	4	0.1	3.67E-03
GO:0009051	pentose-phosphate shunt, oxidative branch	7	4	0.11	5.26E-03
GO:0007606	sensory perception of chemical stimulus	12	6	0.19	7.98E-04
GO:0006098	pentose-phosphate shunt	15	6	0.24	1.12E-03
GO:0051156	glucose 6-phosphate metabolic process	16	6	0.26	1.02E-03
GO:0006740	NADPH regeneration	16	6	0.26	7.63E-04
GO:0007600	sensory perception	17	6	0.27	8.16E-04
GO:0006739	NADP metabolic process	19	6	0.31	1.16E-03
GO:0050877	nervous system process	20	6	0.32	1.28E-03
GO:0003008	system process	21	6	0.34	1.42E-03
GO:0044275	cellular carbohydrate catabolic process	14	4	0.23	4.17E-02
	Molecular Function				
GO:0004616	phosphogluconate dehydrogenase (decarboxylating) activity	10	6	0.16	1.72E-04
GO:0016616	oxidoreductase activity, acting on the CH-OH group of donors, NAD or NADP as acceptor	51	7	0.82	2.80E-02
GO:0016614	oxidoreductase activity, acting on CH-OH group of donors	57	7	0.92	3.57E-02
